# Successful Renal Transplantation after Presumed Cyanide Toxicity Treated with Hydroxocobalamin and Review of the Literature

**DOI:** 10.1155/2018/3753479

**Published:** 2018-09-09

**Authors:** Ryan J. Hendrix, Paulo N. Martins, Jeffrey S. Stoff, Aaron Ahearn, Adel Bozorgzadeh, Babak Movahedi

**Affiliations:** ^1^University of Massachusetts Medical School, Department of Surgery, Division of Organ Transplantation, USA; ^2^University of Massachusetts Medical School, Department of Medicine, Division of Nephrology, USA

## Abstract

We report two cases of successful renal transplantation with allografts from donors who suffered anoxic brain injury as the primary cause of death from house fires. Each was treated prophylactically with hydroxocobalamin (Cyanokit) for suspected cyanide toxicity. During organ procurement, gross examination was notable for deep discoloration of the parenchymal tissues. Approximately 6 and 18 months after transplantation, both recipients have excellent renal graft function and remain independent from hemodialysis (HD). Hydroxocobalamin is the antidote for suspected acute cyanide toxicity. While largely tolerated by the recipient, there is concern over the potential functional implications of the associated side effects of dramatic tissue discoloration and development of oxalate crystals. Furthermore, difficulties performing hemodialysis in patients treated with hydroxocobalamin have been reported due to discoloration of the effluent fluid impacting the colorimetric sensor, causing false alarms and repetitive interruptions. As such, many transplant centers in the United States (US) continue to reject these organs. We seek to highlight two cases of successful transplantation following donor administration of hydroxocobalamin (Cyanokit) and present the first documented case of successful perioperative intermittent hemodialysis following transplantation of an allograft exposed to hydroxocobalamin. Furthermore, we emphasize the importance of optimal organ utilization and caution against unnecessary refusal.

## 1. Introduction

End stage renal disease (ESRD) is a devastating medical condition representing the most advanced form of chronic kidney disease (CKD). Five-year survival rates for patients undergoing renal transplantation are 85.5%, while those on hemodialysis (HD) are significantly reduced at 35.8% [1–3]. Of the 10,286 deceased donor kidney transplant operations performed in 2017, asphyxiation was the documented mechanism of death for 6% of donors [2]. While the etiology of asphyxia is multifactorial and diverse, house fires are a major contributor [2, 4].

Annually, there are approximately 16,000 domestic fire related injuries and 3,000 deaths [4]. Greater than 75% of these fatalities are directly attributed to smoke inhalation toxicity from carbon monoxide (CO) and hydrogen cyanide (CN) gases [4–6]. CN can be liberated during the combustion of products containing both carbon and nitrogen, notably wool, silk, polyurethane (insulation/upholstery), polyacrylonitriles (plastics), melamine resins (household goods), and synthetic rubber [5, 6].

Cyanide is an extremely toxic poison. Mechanistically, cyanide binds rapidly with cytochrome a3, a component of the cytochrome c oxidase complex in mitochondria. Inhibition of cytochrome a3 prevents the cell from using oxygen and forces anaerobic metabolism, resulting in lactate production, cellular hypoxia and metabolic acidosis [7, 8]. Untreated, this cascade can lead to death within minutes. The presence and extent of cyanide poisoning are often initially unknown given the absence of a rapid, confirmatory cyanide blood test. Thus, treatment decisions must be made on the basis of clinical history, mechanism of injury, and suspicion of cyanide intoxication.

The antidote, hydroxocobalamin, classically induces an abnormal reddish discoloration of renal allografts, and oxalate crystals on histologic examination are common. Despite these disconcerting physical features, no evidence suggests an effect on short- or long-term renal function. The only issue lies in the potential need for perioperative HD following renal transplantation. Discoloration of the dialysate can lead to altered refractive properties as detected by the HD machine photodetector. Ultimately, this results in the repetitive triggering of the blood leak alarm initiating a cascade which halts the blood pump and pauses the dialysis cycle.

Here we present two cases of successful renal transplantation utilizing organs that were severely discolored due to prior administration of hydroxocobalamin to the donor and therefore declined by the majority of other centers in our region. For both cases, our standard institutional protocols were followed which specify all patients receive depleting antibodies as part of their induction regimen. Patients that are considered immunologically high risk such as those receiving a second transplant, the presence of donor specific antibodies (DSA), or high calculated panel reactive antibodies (cPRA) receive anti-thymocyte globulin. Low immunologic risk patients receive alemtuzumab.

## 2. Case 1

**Figure 1:**
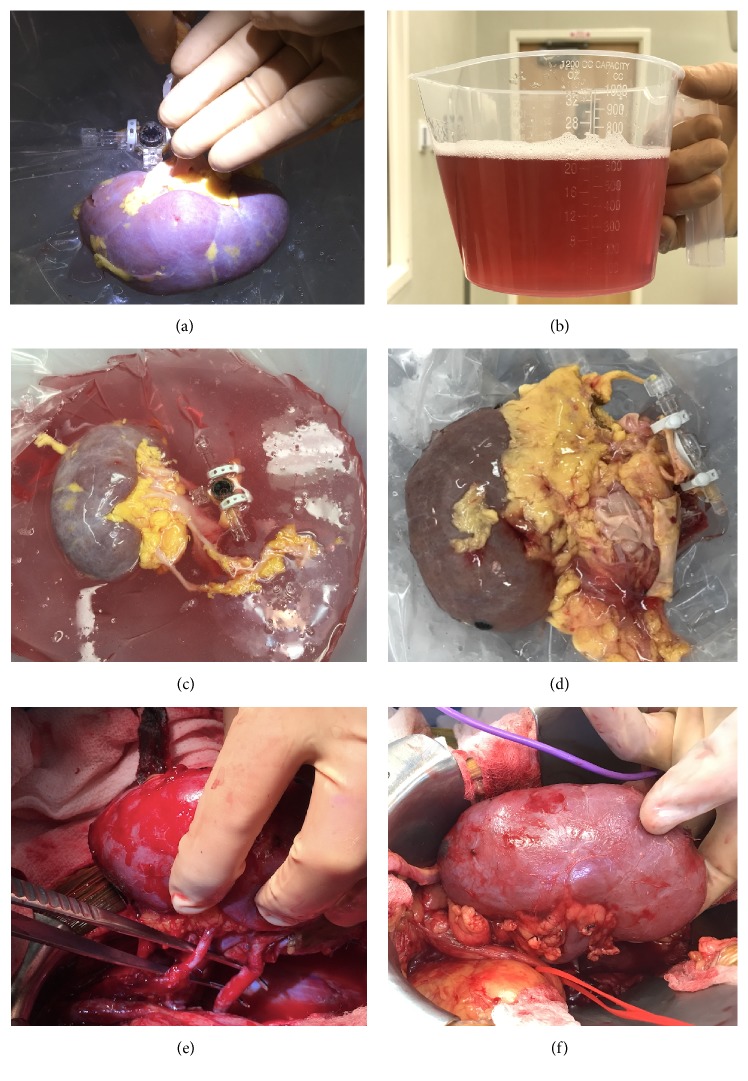
(a) Allograft #1 at time of procurement. (b) Discoloration of effluent. (c) Allograft #1 on pump. (d) Allograft #2 on pump. (e) Allograft #1 in vivo. (f) Allograft #2 in vivo.

A 58-year-old female was found unresponsive after a house fire, with the primary cause of death anoxic brain injury. Despite a short period of asystole, her renal function was preserved with an admission creatinine of 0.88, peak creatinine of 1.24, and a final creatinine of 1.04. The Kidney Donor Profile Index (KDPI) was 64%. On gross examination, severe organ discoloration was noted (Figure 1), and biopsy results were significant for the presence of oxalate crystals. Kidney pump pressure was set at 35 mmHg, resulting in a flow of 133 mL/min with a resistance of 0.23 (Table 1). The organ recipient was a 53-year-old male with end stage renal disease secondary to hypertensive nephrosclerosis on hemodialysis three times per week for a total duration of 52 months. His cPRA was 0% with no evidence of gray zone DSA (Table 2). A complement dependent cytotoxicity (CDC) T cell cross match was performed and negative.

Following transplantation, induction immunosuppression consisted of alemtuzumab 30 mg and solumedrol 500 mg, followed by a maintenance regimen of tacrolimus, mycophenolate 500mg twice daily and prednisone 10 mg daily. Early in the perioperative period, the patient's hospital course was complicated by severe hyperkalemia and delayed graft function requiring HD on postoperative day (POD) 1, 2, 4, and 6. Each session was performed without incident. On POD 7, in the setting of ongoing oliguria and worsening abdominal distention, laboratory and imaging studies revealed a large pelvic fluid collection consistent with an acute perinephric hematoma. The patient was taken emergently to the operating room for reexploration and evacuation of the hematoma. Subsequently, the patient's urine output began to improve and serum creatinine started to decrease. Ultimately, the patient was discharged to home on POD 13.

**Figure 2:**
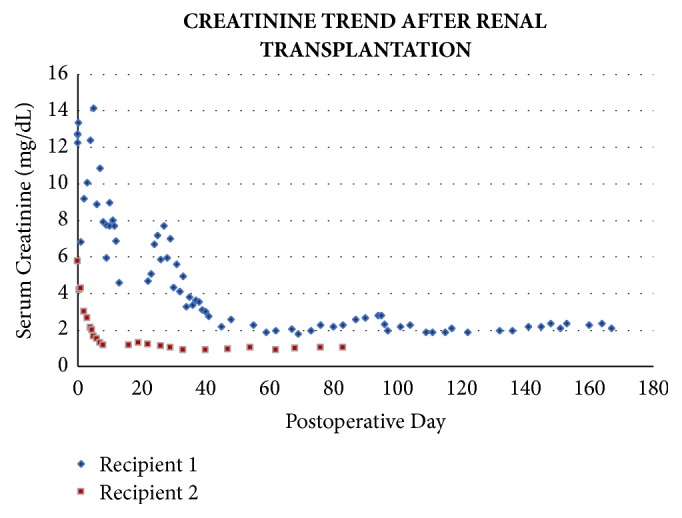
Serum creatinine (mg/dL) following renal transplantation. Above demonstrates the significant improvement in renal function after renal transplantation and the sustained viability of each organ.

Approximately 3 weeks after transplant, the patient presented with an elevated creatinine to 4.70 mg/dL (previous nadir 2.60 mg/dL). A biopsy of the renal allograft revealed acute T cell mediated rejection (Banff category 2A) as well as acute antibody-mediated rejection. A C4D stain was positive in more than 30% of the peritubular capillaries. A Human Leukocyte Antigen (HLA) antibody screen revealed a class II (DQ) DSA at 8000 mean fluorescence intensity (MFI). Institutional protocols for rejection were followed, and the patient was initially treated with solumedrol 500 mg x3 doses. Despite this, his creatinine continued to rise and he remained oliguric. Subsequently, anti-thymocyte globulin 1 mg/kg x 3 doses was administered, followed by plasmapheresis and IVIG x 5 doses (2g/kg ideal body weight). During this 2-week hospital readmission for rejection, 4 additional sessions of HD were required, and again, there were no complications. A repeat biopsy 6 weeks later did not reveal any signs of ongoing rejection, and all prior oxalate crystals had disappeared. The patient's creatinine improved and plateaued in the 1.9-2.0 range. Eighteen months after transplant, the patient has a stable graft function with creatinine levels near 2 mg/dL (Figure 2).

## 3. Case 2

A 57-year-old male found unresponsive after a house fire subsequently developed pulseless electrical activity (PEA) arrest with the primary cause of death documented as anoxic brain injury. The donor met criteria to be considered public health services (PHS) high risk given his recent history of incarceration. Overall, renal function was preserved with a creatinine on admission of 1.60, peak creatinine of 1.60, and final creatinine of 1.40. The KDPI was 75%. On gross examination, severe organ discoloration was noted (Figure 1), however, renal biopsy did not reveal the presence of oxalate crystals. Kidney pump pressure was set at 35 mmHg, resulting in a flow of 94 mL/min with a resistance of 0.30 (Table 1). The organ recipient was a 78-year-old male with ESRD secondary to hypertension and diabetes undergoing HD three times a week for a total duration of 25 months. The cPRA was 0%, however, a single gray zone DSA against HLA A29 was identified (Table 2). All crossmatch tests were negative.

Induction immunosuppression consisted of anti-thymocyte globulin (total dose of 5mg/kg) administered over 5 doses and solumedrol 500 mg, followed by a maintenance regimen of tacrolimus, mycophenolate 500 mg twice daily and prednisone 10 mg daily. The patient had an overall uneventful postoperative course with immediate graft function and progressive downtrend in creatinine levels. On POD 7, the patient was discharged to a short-term rehabilitation facility. Six months after organ transplantation, the patient continues to experience a stable graft function with baseline creatinine of 1.14 (Figure 2).

## 4. Discussion

Cyanide is a lethal toxin which uncouples mitochondrial oxidative phosphorylation; preventing aerobic metabolism and initiating a cascade of acidosis and hypoxia. Hydroxocobalamin, the gold standard treatment for cyanide toxicity, was FDA-approved in the United States in 2006 but has been formally licensed in Europe since 1996 to treat known or suspected cyanide poisoning [9]. The initial dose of hydroxocobalamin for adults is 5 grams administered as an intravenous (IV) infusion over fifteen minutes. Intravenous administration of hydroxocobalamin leads to interaction and binding to plasma proteins and low molecular weight physiologic compounds generating various cobalamin-(III) complexes. These molecules can then bind cyanide ions through a substitution for the hydroxo ligand linked to the trivalent cobalt ion. End product is cyanocobalamin, which is then excreted in the urine.

Pharmacodynamic effects include an increase in blood pressure and variable effects on heart rate. Dose-proportional pharmacokinetics has been observed following administration of hydroxocobalamin with total urinary excretion calculated to be 60-70% of a given dose of hydroxocobalamin. Discoloration of urine has been noted to occur for up to 35 days after administration. Mean half-life of free cobalamins-(III) was 26 hours at the 5g dosage. Most common adverse reactions include chromaturia, erythema, and oxalate crystal formation. These crystals have been observed in the urine of both healthy subjects given hydroxocobalamin and patients treated with hydroxocobalamin following suspected cyanide poisoning [10]. Interestingly, oxalate crystals were observed in donor 1's kidney biopsy performed at the time of organ recovery. Most patients will experience a reversible red/orange discoloration of the skin, urine and mucous membrane secretions which can contribute to alterations in colorimetric tests and co-oximetry measurements [11, 12].

The first published case report of a successful organ transplant from a donor, whose cause of death was cyanide toxicity, was published in 1987 [13]. Several additional case reports and small series echoed the findings that cyanide toxicity itself was not a contraindication to transplant [14–17]. Since 2006, hydroxocobalamin has established itself as the gold standard antidote for cyanide toxicity replacing amyl nitrate and sodium thiosulfate. Initial reports suggested Cyanokit administration was safe prior to organ transplantation [18]. However, more recent reports have found hydroxocobalamin to be associated with failure of intermittent hemodialysis, thus limiting the opportunity to provide life-saving treatment should a transplanted allograft fail or experience delayed function [19, 20]. The presumed mechanism for this phenomenon is the discoloration of bodily fluids triggering a “pseudo-blood leak” [21]. This triggering of the colorimetric sensor of the dialysis machine subsequently causes false alarms and repetitive interruptions of the dialysis cycle.

While it is not possible to determine the exact reasoning for neighboring transplantation centers to decline the organs, discussions with the Organ Procurement Organization (OPO) highlighted genuine concern regarding the feasibility of perioperative dialysis. A recent study postulated that hemodialysis machines employing a photodetector consisting of a single optical emitter designed to detect light scatter and signal drop off are unlikely to be affected by hydroxocobalamin whereas those using a dual LED array that depends on light absorption are susceptible [22, 23]. In congruence with this explanation, a multidisciplinary discussion was held at our institution prior to transplantation at which time it was confirmed the dialysis machine leak alarm could be overridden should dialysis be required.

## 5. Conclusion

Although we present only two cases, our experience reiterates that kidneys from donors treated with hydroxocobalamin can be used with good long-term outcome. Furthermore, we present the first documented case of successful perioperative intermittent HD following transplantation of an allograft exposed to hydroxocobalamin.

## Figures and Tables

**Table 1:** Donor demographics and organ parameters.

	Donor 1	Donor 2
Age (yrs)	58	57
Sex	Female	Male
Weight (kg)	94.4	60.8
KDPI^*∗*^ (%)	64	75
Cause of Death	Anoxia	Anoxia
Mechanism	House Fire	House Fire
Creatinine (mg/dL)		
First	0.88	1.60
Peak	1.24	1.60
Last	1.04	1.40
Biopsy at Procurement		
Glomerulosclerosis (%)	0	1
Tubular Fibrosis (%)	1-10	1-10
Vessel Atherosclerosis	Absent	Mild
Oxalate Crystals	Positive	Negative
Serology		
CMV	Negative	Positive
EBV	Negative	Positive
HBV	Negative	Positive
HCV	Negative	Negative
HIV	Negative	Negative
NAT^*∗∗*^, HBV, HCV, HIV	Negative	Negative
PHS^*∗∗∗*^ Increased Risk	Negative	Positive
Final Pump Parameters		
Pressure (mmHg)	35	35
Flow (mL/min)	133	94
Resistance	0.23	0.30
Organ Laterality	Right	Right

^*∗*^KDPI: Kidney Donor Profile Index.

^*∗∗*^NAT: Nucleic Acid Testing.

^*∗∗∗*^PHS: U.S. Public Health Service.

**Table 2:** Recipient demographics.

	Recipient 1	Recipient 2
Age (yrs)	53	78
Sex	Male	Male
Disease Etiology	Hypertension	Type II Diabetes
Dialysis Duration (months)	52	25
Sequence Match	9	470
cPRA^*∗*^ (%)	0	House Fire
Gray zone Antibody	Negative	A29
CMV	Negative	Negative
EBV	Positive	Positive
HBV/HCV	Negative	Negative
Cold Ischemia Time (hrs)	22	21.65
Warm Ischemia Time (mins)	48	25

^*∗*^cPRA: Calculated Panel Reactive Antibody.
